# Comparison of Hydroxyethyl starch 130/0.4 (6%) with commonly used agents in an experimental Pleurodesis model

**DOI:** 10.1186/s12890-020-01260-1

**Published:** 2020-08-27

**Authors:** Hasan Oğuz Kapicibasi, Hasan Ali Kiraz, Nazli Demir Gök

**Affiliations:** 1 Department of Thoracic Surgery, Canakkale Onsekiz Mart University, Faculty of Medicine, Canakkale, Turkey; 2 Department of Anesthesiology, Canakkale Onsekiz Mart University Faculty of Medicine, Canakkale, Turkey; 3 Department of Pathology, Izmit Seka State Hospital, İzmit, Kocaeli Turkey

**Keywords:** Parietal-visceral pleura, Pleurodesis, Hydroxyethyl starch

## Abstract

**Background:**

Hydroxyethyl Starch (HES) 130/0.4 (6%) is a commonly used intravascular volume expander with anti-inflammatory and antioxidant properties. In this study, we aimed to compare the histopathologic activity of HES 130/0.4 (6%) with various widely-used agents in pleurodesis.

**Methods:**

Forty male Wistar-Albino rats were divided into five groups: controls, povidone-iodine recipients (PI group), sterile talcum recipients (Talcum group), autologous blood recipients (AB group) and HES 130/0.4 (6%) recipients (HES group). Thirty days after application of agents, pleural and lung tissues were resected. Evaluation was performed via macroscopic scoring (adhesion) and specimens were stained with H&E for microscopic examination (inflammation and fibrosis).

**Results:**

HES recipients had significantly higher adhesion compared to controls (lower grade 0, higher grade 1 frequency vs. controls), they were found to have significantly lower frequency of grade 2 adhesion (vs. PI, Talc and AB) and grade 3 adhesion (vs. AB), indicating that the adhesion-generating properties of HES were only superior to the control group. HES recipients had significantly higher inflammatory grades compared to controls (lower grade 0, higher grade 1 frequency), while they had lower grades compared to the PI, Talc and AB groups. Although the PI, Talc and AB groups were statistically similar in most comparisons, we observed a trend towards higher success with the use of Talc and especially AB.

**Conclusion:**

Our results do not support a role for HES in pleurodesis. We believe that the autologous blood method remains as an effective and successful procedure without side effects.

## Background

Pleurodesis is a procedure in which the anatomical space between the parietal and visceral pleura is removed by the adhesion of these two layers. While adhesion can be achieved mechanically by abrasion via thoracoscopy or thoracotomy, chemical pleurodesis is very common with the application of an agent via thoracoscopy and chest tube insertion. The most frequently used agent in the latter approach to pleurodesis is talcum powder, also known as talc [1]. However, when the research concerning pleurodesis methods is evaluated, it is evident that there is no “ideal” agent for pleurodesis [2], and success rates with different agents vary greatly, from 54 to 93% [3].

An ideal agent for chemical pleurodesis should be easy to apply, effective, accessible, inexpensive and must have minimal side effects. Due to these prerequisites, the literature has focused on the use of autologous blood (AB) and povidone-iodine (PI) as alternatives for chemical pleurodesis [4–6]. Although talc pleurodesis is still very common, reports have shown possibility of serious adverse effects, including acute respiratory distress [7–9]. Hydroxyethyl starch (HES) 130/0.4 (6%) (Voluven®, Fresenius Kabi, Germany) is a corn-based intravascular volume expander that is readily used during the perioperative period and in intensive care units. Studies have shown that this colloid solution has antioxidant and anti-inflammatory properties, which may be associated with its positive contribution to ventilation and oxygenation [10]. Moreover, HES has been reported to be associated with a reduction in the need for blood transfusion in major surgical procedures [11].

Epidural blood patch application is considered as the gold standard approach in patients unresponsive to symptomatic treatment; however, in cases where the use of autologous blood is contraindicated, the introduction of HES 130/0.4 (6%) into the epidural space has been reported as a suitable alternative [12], indicating that HES may be a safe and effective option in other scenarios. In the lung, AB patches are utilized in clinical and experimental studies with considerable success for various conditions. Therefore, evaluation of the efficacy of HES in chemical pleurodesis may be important to identify alternative methods.

This study aimed to compare and evaluate the effectiveness of the corn-based, easy-accessible volume expander HES 130/0.4 (6%) with other widely-used agents for pleurodesis, in terms of efficacy and histopathological outcomes.

## Methods

Forty male Wistar-Albino rats suitable for the study conditions, weighing 350–450 g, were included in the study. Ethical approval was obtained from the Canakkale Onsekiz Mart University Ethical Board of Animal Studies (2018/1800097318). All rats were subjected to a general clinical examination of behavior and respiratory and cardiovascular characteristics at the beginning of the study and on a weekly basis after interventions by a member of the research staff (excessive pain symptoms, changes in behavior, activity). Additionally, the animals were regularly followed by veterinarians who were staffed at the animal studies laboratory. Rats were kept in appropriate-sized cages and were fed ad libitum with standard rodent chow during the course of the study. Ambient temperature was set at 21 ± 2 C degrees and a normal daily light cycle was simulated by 12 h of light and dark. All rats were cared for in accordance with the “Regulation on the Welfare and Protection of Animals Used for Experimental and Other Scientific Purposes (13.12.2011-28141)” prepared by the Ministry of Food, Agriculture and Livestock.

**Fig. 1:**
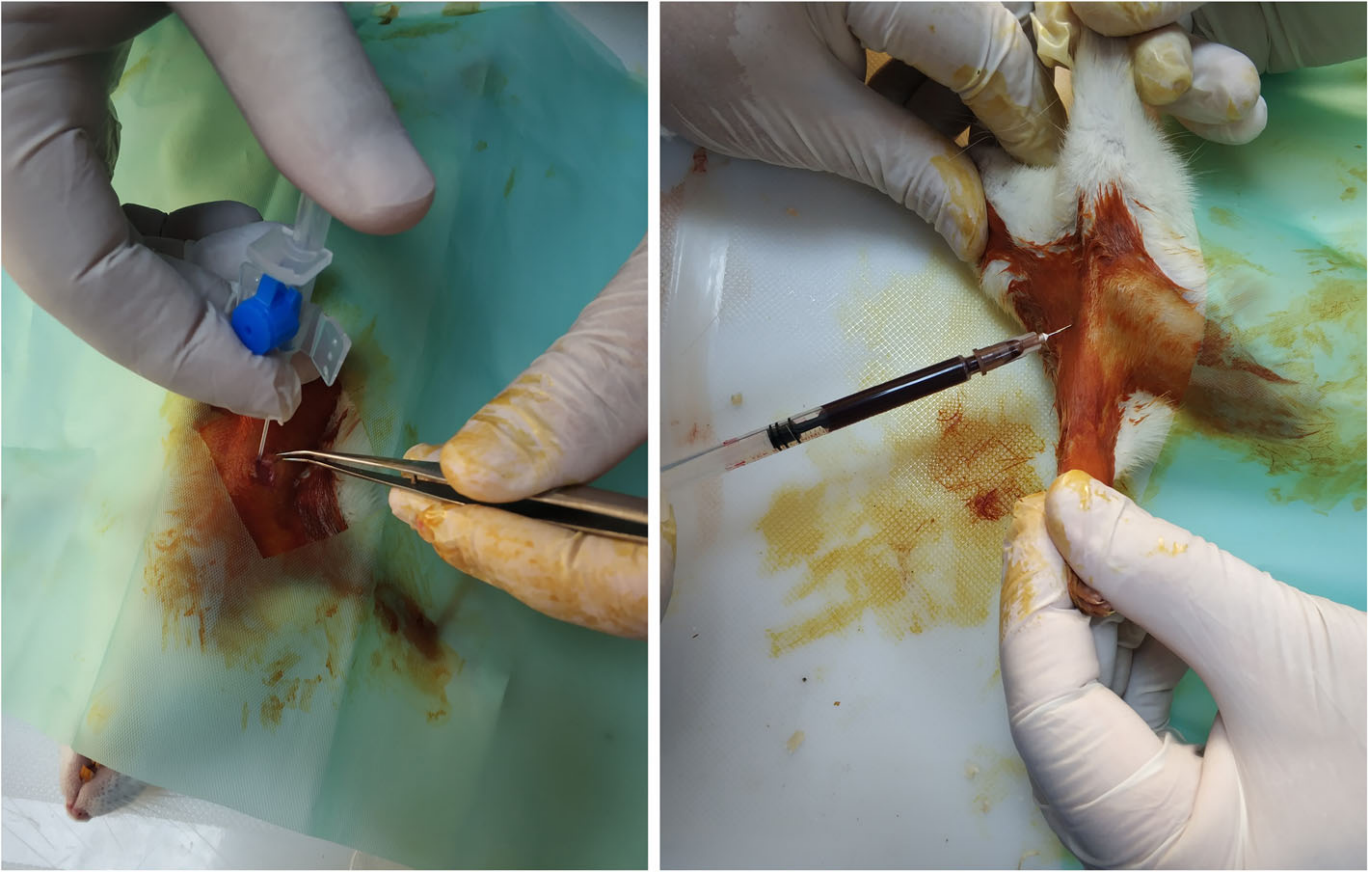
The surgical procedure. Cannulation (**a**) and blood tapping (**b**) from the subclavian vein for autologous pleurodesis

After the adaptation period, forty rats were randomly allocated into five groups of eight rats each:

- Controls (sham control group, *n* = 8): This was the control group which underwent the same procedures but received intrapleural 2 mL/kg physiological saline (SF).
- Povidone-iodine group (PI group, *n* = 8): Rats in this group were administered intrapleural 2 mL/kg povidone-iodine (%10) [13–15].
- Sterile talcum group (Talc group, *n* = 8): Rats in this group received intrapleural 2 mL/kg sterile talcum.
- Autologous blood group (AB group, *n* = 8): Rats in this group were administered intrapleural autologous blood obtained from the subclavian vein (Fig. 1) at a dose of (2 mL/kg) as described by Özpolat and colleagues [16].
- Hydroxyethyl starch (130/0.4, 6%) (HES group, *n* = 8): Rats in this group were administered intrapleural corn-based HES 130/0.4 (6%) 2 mL/kg.

### Surgical procedures

Intrapleural administration was performed in all groups. After necessary skin preparation and following general anesthesia (xylazine - 5 mg/kg and ketamine - 50 mg/kg, intramuscular), a 3–5 mm skin incision was made under the 5th intercostal space of the right hemithorax under sterile conditions. Using a 22-Gauge polytetrafluoroethylene (PTFE) catheter, agents were applied to the pleural space with the aid of a three-way tap. (Fig. 1).

The presence of air in the pleural space was controlled. If air was present in the pleural cavity, it was removed with the help of the a triple tap. The pleural catheterization was then terminated and the skin was sutured. The rats were then slowly rotated to enable the spread of agents to the entire pleura. All interventional procedures were carried out from 8 AM to 11 AM.

**Fig. 2:**
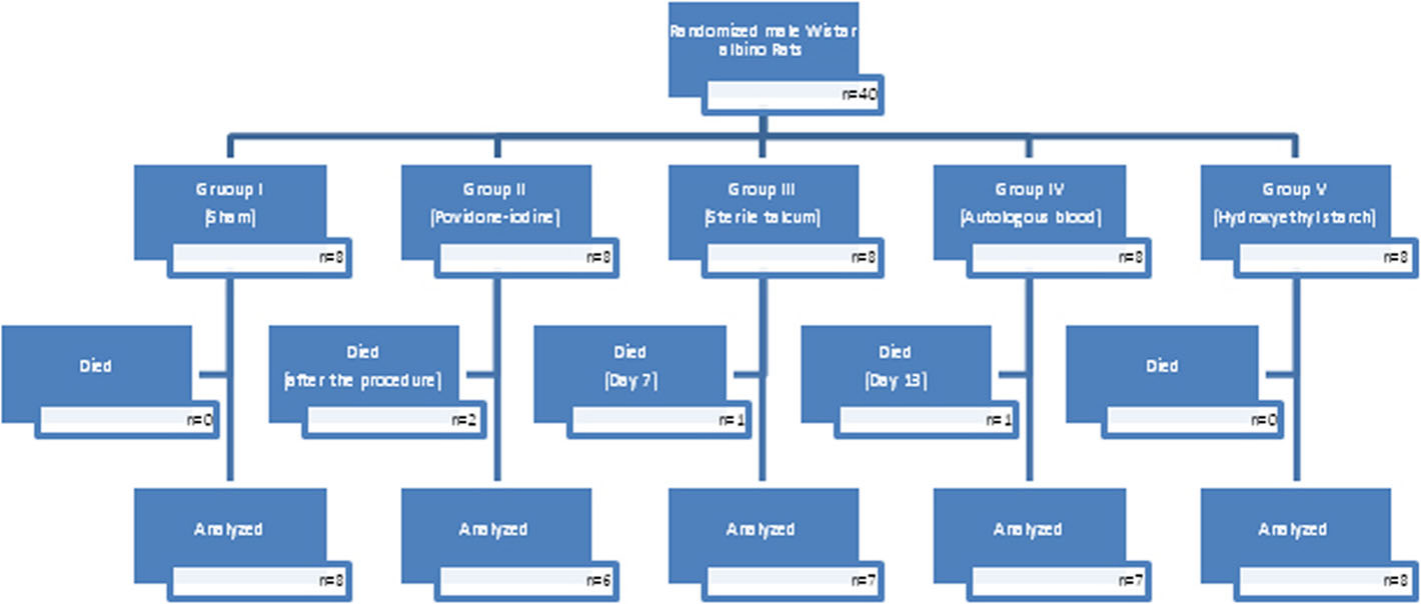
Study flow chart

Two rats from the PI group died immediately after the procedure. One rat from the sterile talc and one from autologous blood groups were lost on the 7th and 13th days due to surgical complications (Fig. 2).

Thirty days after the procedures, the rats were sacrificed via administering high dose anesthetic (xylazine - 10 mg/kg and ketamine - 80 mg/kg, intramuscular), right hemithorax ribs were cut from sternal junctions, and the pleural spaces were exposed. Pleural and lung tissue sampling was performed after macroscopic scoring in all groups. The macroscopic scoring of pleurodesis was determined by a surgeon and a blinded pathologist according to the method described by Hurewitz et al. [17]: Grade 0 = Normal pleura; Grade 1 = Adhesions at several sites, Grade 2 = Widely dispersed adhesion, and Grade 3 = Complete adherence to the pleural space. The specimens were then fixed with formalin.

### Histopathological analysis

The obtained lung tissue samples were fixed in 10% formalin solution. After 24 h of fixation, the tissues were transferred to cassettes according to the groups. The tissues were then embedded in paraffin and sections of 4-μm thickness were taken for hematoxylin & eosin (H&E) staining. The resultant samples were examined under a light microscope. The examining pathologists were blinded to the groups. The peribronchial, perivascular, and parenchymal inflammatory cell infiltration and fibrosis findings in the tissues were scored and classified categorically according to the degree of inflammation (Grade 0: none, Grade 1: > 0–5%, Grade 2: > 5–30% = 2, and Grade 3: > 30%).

### Statistical analysis

The applied method and the minimum number of animals required for scientific results were taken into consideration with regard to the reports of previous studies which had utilized groups comprising of 6 to 10 animals [16, 18–20]. With this data in mind, we used the values reported by Ozpolat et al. (microscopic scoring results with 2 ml/kg AB application, mean ± SD = 1.86 ± 0.69) and determined a 50% change in mean value to be significant. This study was chosen because it had utilized AB which is a safe and frequently used approach, also it had relatively low standard deviation compared to other studies. The number of animals required for the comparison of groups was calculated as 8 –with 80% power and an alpha error of 5%. The Statistical Package for the Social Sciences (SPSS) version 25.0 software (SPSS Inc., Chicago, IL, USA) was used for data analysis. The groups were compared with the Pearson Chi-square test; subset comparisons were performed via the use of Z-score comparisons for column proportions. No correction was made in terms of multiple hypothesis testing. *P*-values of ≤0.05 were accepted as significant.

## Results

After the conduct of interventions, two rats in the PI group had died; thus, analysis was performed with 6 rats in this group. Additionally, one rat each from the Talc and AB groups died (on the 7th and 13th days after intervention, respectively), reducing the total rat count in these groups to 7 rats each. All remaining animals were included in the analyses, since none were excluded or euthanized prematurely during follow-up evaluations.

### Macroscopic findings

**Fig. 3:**
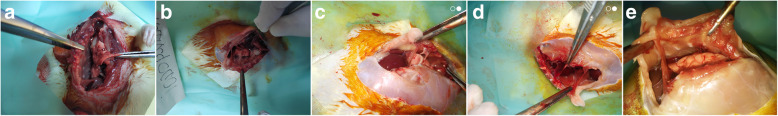
Macroscopic appearances in the different groups. **a** Macroscopic appearance in the SF group. **b** Macroscopic appearance in the povidone-iodine group. **c** Macroscopic appearance in the autologous pleurodesis group. **d** Macroscopic appearance in the sterile talcum group. **e** Macroscopic appearance in the HES 130/0.4 (6%) group

Overall comparison of adhesion grades in the 5 groups showed a statistically significant difference between groups (Pearson Chi-square, *p* < 0.001). In the control group, all rats were classified as “normal pleura” (grade 0). Grade 2 and 3 adhesions were significantly more common in the AB group compared to the HES group and controls. Although there was no significant difference between the frequency of grade 2 and 3 adhesions in the comparison of the PI, Talc and AB groups, we observed a trend towards higher frequency of grade 3 adhesion in the AB group (42.9%) compared to the Talc (14.3%) and PI (0%) groups (Table 1). Although HES recipients had significantly higher adhesion compared to controls (lower grade 0, higher grade 1 frequency vs. controls), they were found to have significantly lower frequency of grade 2 adhesion (vs. PI, Talc and AB) and grade 3 adhesion (vs. AB), indicating that the adhesion-generating properties of HES were only superior to the control group. Of note, 3 subjects from the AB group (42.9%) and 1 subject from the Talc group (14.3%) had complete adherence of the pleura. When the contralateral pleural surfaces were evaluated, we found no adhesions in the parietal, visceral, and mediastinal surfaces (Fig. 3).

**Table 1:** Comparison of pleural adhesion levels between the experimental groups

	Control (*n* = 8)		PI (*n* = 6)		Talc (*n* = 7)		AB (*n* = 7)		HES (*n* = 8)	
	n	%	n	%	n	%	n	%	n	%
Normal pleura	8_a_	100	0_b_	0	0_b_	0	0_b_	0	4_c_	50
Adhesions at several sites	0_a_	0	2_a, b_	33.3	0_a_	0	0_a_	0	4_b_	50
Widely dispersed adhesion	0_a_	0	4_b_	66.7	6_b_	85.7	4_b_	57.1	0_a_	0
Complete adherence	0_a_	0	0_a, b_	0.0	1_a, b_	14.3	3_b_	42.9	0_a_	0

Pearson Chi-square test with subset comparison for column proportions. Chi-Square = 48.816, *p* < 0.001. Percentages represent column proportion. The same letters indicate the lack of significant difference from other group subsets (No correction for multiple hypothesis testing)

### Histopathological findings

Overall comparison showed a statistically significant difference in terms of the distribution of inflammation grades in the study groups (Pearson Chi-square, *p* < 0.001). The distributions within groups were similar to those observed with adhesion (macroscopic characteristics). Seven of the subjects in the control group had no inflammation and one subject had grade 1 inflammation. In the HES group, 3 (37.5%) had grade 0 and 5 (62.5%) had grade 1 inflammation. Comparisons revealed that HES recipients had significantly higher inflammatory grades compared to controls (lower grade 0, higher grade 1 frequency), while they had lower grades compared to the PI, Talc and AB groups. There were no statistically relevant differences between the PI, Talc and AB groups in terms of inflammatory grade distribution; however, we again observed a trend towards higher levels of inflammation (grade 2 + 3) in the AB group (*n* = 5, 71.4%) compared to the PI group (*n* = 3, 50%) and the Talc group (*n* = 4, 57.1%). Of note, in the whole study group (*n* = 36) only the AB group had grade 3 inflammation (*n* = 2) (Table 2).

**Table 2:** Comparison of inflammation levels between the experimental groups

	Control (*n* = 8)		PI (*n* = 6)		Talc (*n* = 7)		AB (*n* = 7)		HES (*n* = 8)	
	n	%	n	%	n	%	n	%	n	%
Non-existent	7_a_	87.5	0_b_	0	0_b_	0	0_b_	0	3_b_	37.5
Inflammation between > 0–5%	1_a_	12.5	3_a. b_	50	3_a. b_	42.9	2_a. b_	28.6	5_b_	62.5
Inflammation between > 5–30%	0_a_	0	3_b_	50	4_b_	57.1	3_b_	42.9	0_a_	0.0
Inflammation between > 30–100%	0_a_	0	0_a_	0	0_a_	0	2_a_	28.6	0_a_	0.0

Pearson Chi-square test with subset comparison for column proportions. Chi-Square = 35.633, *p* < 0.001. Percentages represent column proportion. The same letters indicate the lack of significant difference from other group subsets (No correction for multiple hypothesis testing)

When fibrosis was evaluated, overall comparison demonstrated the presence of a statistically significant variation in the distributions of groups (Pearson Chi-square, *p* < 0.001). All subjects in the control (*n* = 8) and HES (*n* = 8) groups were classified as “no fibrosis”, indicating no difference between HES and controls in terms of fibrosis. Statistical results only showed that the PI, Talc and AB groups had significantly higher levels of fibrosis in comparison to the control and HES groups. There was no significant difference between the PI, Talc and AB groups with regard to the distribution of fibrosis classifications; however, fibrosis was present in 95% of the subjects in these 3 groups (19/20). Only 1 subject in the Talc group was classified as “no fibrosis” (Table 3).

**Table 3:** Comparison of fibrosis levels between the experimental groups

	Control (*n* = 8)		PI (*n* = 6)		Talc (*n* = 7)		AB (*n* = 7)		HES (*n* = 8)	
	n	%	n	%	n	%	n	%	n	%
No fibrosis (0%)	8_a_	100	0_b_	0	1_b_	14.3	0_b_	0	8_a_	100
Fibrosis between > 0–5%	0_a_	0	6_b_	100	4_b_	57.1	5_b_	71.4	0_a_	0
Fibrosis between > 5–30%	0_a_	0	0_a_	0	2_a_	28.6	2_a_	28.6	0_a_	0

Pearson Chi-square test with subset comparison for column proportions. Chi-Square = 36.928, *p* < 0.001. Percentages represent column proportion. The same letters indicate the lack of significant difference from other group subsets (No correction for multiple hypothesis testing)

**Fig. 4:**
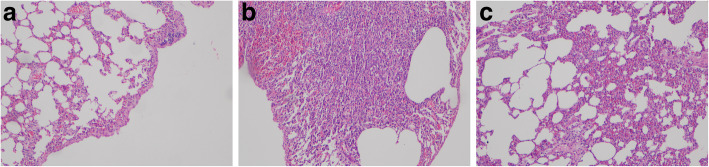
Microscopic evaluations in the different groups. **a**: Sterile Talcum group; Development of fibrosis in the visceral pleura (arrow) and emphysematous changes in the subpleural area. H&E, × 100. **b**: Autologous blood transfusion group; Development of mild fibrosis in the visceral pleura around intense parenchymal inflammation areas (arrow). H&E, × 100. **c**: HES 130/0.4 (6%) group; Intense inflammatory response and mild emphysematous changes, H&E, × 100

Evaluation of contralateral surfaces showed that both inflammation and fibrosis were absent from the parietal, visceral and mediastinal pleural surfaces, as well as the lung and diaphragm (Fig. 4).

## Discussion

Historically, various agents (PI, minocycline, tetracycline, OK 432, erythromycin, and Talc) have been used to induce pleurodesis [21]. Today, the most popular of these agents are arguably PI, Talc and AB. Our study was aimed at comparing HES with these agents in terms of pleurodesis development. We also performed a detailed review of notable publications using these three agents in comparable animal studies (rats and rabbits) to identify study characteristics, dosage and toxicity of these agents (Table 4).

**Table 4:** Detailed evaluation of previous experimental animal studies (rats and rabbits) of pleurodesis

Agent	Study	Species	Dosage	Duration	Toxicity	Notes
Povidone-iodine	Teixeira et al. 2013 [15]	New Zealand rabbits (2–3 kg)	2 ml @ 2, 4 and 10%	1, 3, 7, 14, 28 days	No adverse effects despite relatively high dosage	Macroscopic results showed progressive increase in adhesion with dosage (4 and 10%) and time (demonstrating a plateau after 7 days)
	Lashkarizadeh et al. 2019 [13]	Rats (details not understood)	0.5 ml @ 8%	45 days	No information	Povidone-iodine had similar effectivity with Talc.
	Yazkan et al. 2013 [14]	Wistar-albino rats (260–320 g)	2 ml @ 1, 2 and 4%	30 days	Although 4% dosage demonstrated some microscopic adverse effects in the contralateral pleura, statistical significance was not present.	2 and 4% dosage deemed to be similar in effect, and both were better than 1%. All three concentrations were deemed safe, but 2% was advised.
Talc	Vanucci et al. 2018 [22]	New Zealand rabbits (2.1–2.5 kg)	2 ml @ 40 mg/kg and 200 mg/kg	14 and 28 days	Pleural granulomas were observed with both doses in all subjects on day 14. In the 40 mg/kg group, only 40% of the subjects had granulomas on the 28th day. All 200 mg/kg recipients had granulomas on the 28th day.	Adhesion did not seem to increase with higher dosage, but somewhat increased with time (14 vs. 28 days)
	Gozubuyuk et al. 2010 [23]	Wistar-albino rats (280–320 g)	0.5 ml @ 60 mg/kg	72 h and 7 days	Significant alveolar edema, hemorrhage and inflammation in the acute phase (72 h) compared to tetracycline and controls. At 7 days, edema was also present at a higher frequency.	Results were compared with tetracycline, and showed that Talc caused earlier pleural proliferation and fibrosis.
	Ahn et al. 2015 [18]	Sprague-Dawley rats (220–300 g)	400 mg/kg (volume not reported)	28 days	Talc particles not detected in lung parenchyma. Suggested possibility for systemic effect.	
	Muta et al. 2011 [19]	Wistar rats (300–350 g)	400 mg/kg (volume not reported)	30 days	Talc particles detected in the alveoli.	
	Refosco et al. 2004 [24]	Wistar rats (200–300 g)	2 ml @ 100 mg/kg and 500 mg/kg	45 days	Too few subjects to evaluate toxicity.	No remarkable differences between the two very different doses.
	Mitchem et al. 1999 [20]	New Zealand rabbits (4 kg)	~ 0.25 ml @ 70 mg/kg	30 days	Histological changes in the contralateral lung and blood chemistry changes. Suggested systemic adverse effect.	Although adverse effects were observed, authors concluded Talc was safer than doxycycline (other group).
Autologous Blood	Ozpolat et al. 2010 [16]	Wistar-albino rats (280–310 g)	1 ml/kg, 2 ml/kg and 3 ml/kg	30 days	No microscopic or macroscopic adverse effects in the contralateral pleura and other tissues. No systemic effects.	1 ml/kg did not cause adhesion, 2 ml/kg was effective but 3 ml/kg was deemed more appropriate.
	Yalcinkaya et al. 2019 [25]	Wistar-albino rats (250–300 g)	3 ml/kg	7 and 21 days	At least 1 rat died before the end of the study in each group.	NSAIDs seemed to reduce the formation of pleurodesis when used after the intervention.
	Yildizhan et al. 2016 [26]	Wistar-albino rats (260–320 g)	2 ml/kg	30 days	No inflammation or adhesion in the contralateral pleura, liver or diaphragm. No sign of alveolar injury.	A group which received a 50:50 mix of ozone and 1 ml/kg autologous blood demonstrated better results compared to autologous blood alone (2 ml/kg).
	Mitchem et al. 1999 [20]	New Zealand rabbits (4 kg)	1 ml/kg	30 days	No adverse effects.	No efficacy in contrast to Talc and doxycycline which had significant efficacy.

Talc, tetracycline and doxycycline are agents that are widely used in the treatment of pneumothorax but have potentially devastating side effects, including acute respiratory distress syndrome [22–24]. As a method that did not have similar side effects, AB pleurodesis was first used by Robinson in the treatment of spontaneous pneumothorax [25]. It is relatively safe compared to other forms of chemical pleurodesis, as very few adverse results have been reported [26]. The success rate of AB pleurodesis is reported between 59 and 100% in different publications reporting clinical results [6, 27–30]. Various studies have explored the use of AB in experimental animal studies [31–33]. In an experimental rat study by Ozpolat et al., different volumes of AB application were evaluated for pleurodesis efficacy, and it was reported that 2 and 3 ml/kg volumes were successful, while 1 ml/kg was ineffective [16]. It is also important to note that some animal studies have investigated factors influencing the success of AB pleurodesis. For instance, the use of non-steroid anti-inflammatory medications after the procedure were suggested to reduce efficacy [32], while the addition of ozone to 1 ml/kg AB (50:50) was suggested to increase pleurodesis effectiveness [33]. These findings, although limited, may explain the varying degrees of AB success reported in different clinical studies.

In our experimental study, pleurodesis was best achieved in the Talc and AB groups. Previous studies in animals are often intriguing; however, consistent results have not been reported with any of the methods. Experimental animal studies have frequently utilized Talc as the primary agent to be compared with other candidate agents; however, the majority have reported superior Talc efficacy in both microscopic and macroscopic investigations [18, 19]. The exception to this seems to be doxycycline which demonstrated significant superiority to Talc in a rabbit study by Mitchem and colleagues, albeit with severe local (and possibly systemic) side effects; thus, the authors themselves concluded that preferring Talc over doxycycline would be beneficial [20]. They also found that AB application was not effective in the short term [20]. On the other hand, results with PI in animal experiments seem to be promising. A remarkably detailed rabbit study, comparing PI at 3 different concentrations (2, 4 and 10%), by Teixeira and coworkers, revealed that PI was effective in pleurodesis formation (at 4 and 10% concentrations) in 7 days. They also found that macroscopic findings associated with adhesion had progressively increased with concentration and duration, indicating a somewhat reliable dose-response characteristic. Moreover, no adverse effects were reported in the study [15].

Studies reporting adverse effects with different types of chemical pleurodesis are conflicting. For instance, various concentrations have been utilized for Talc pleurodesis (40–500 mg/kg) with a broad range of study durations (72 h–45 days); however, these studies have failed to determine whether an increase in dosage is associated with the development of pleurodesis or not, even though it is often noted that smaller particle size of Talc may be associated with adverse events [18, 19, 34, 35]. Furthermore, while Gozubuyuk et al. [34] reported significant side effects (including edema and hemorrhage) and Muta et al. [19] detected Talc particles in the alveoli, studies similar in design have not found such results [18, 35]. Our assessment of the contralateral side and other tissues via macroscopic and microscopic analyses did not show any adverse effects with the agents used in the current study. However, biochemical and systemic evaluations were not performed.

In a prospective study of 56 patients with malignant pleural effusion, Keeratichananont et al. compared autologous pleurodesis and talcum powder, and demonstrated that they had an equivalent efficacy. Moreover, they reported that recipients of AB had significantly lower fever, pain score and length of hospital stay [36].

In another clinical study, Ibrahim et al. evaluated 38 patients with malignant pleural effusion in a prospective study comparing PI and Talc for pleurodesis. Their results showed that PI was an effective alternative to Talc [37]. In our study, we observed that PI was not as effective when compared to the AB and Talc groups. However, this observation may be confounded by the loss of 2 rats immediately after the procedure in the PI group. It is well known that PI causes contact dermatitis, allergic reactions, chemical burns, and irritant skin lesions [38]. In another study, Cheong et al. [39] observed that PI causes an initial inflammatory phase with edema, alveolar rupture, and leukocyte infiltration into the pulmonary interstitium. Even though previous studies have not reported significant side-effects with PI [15], we believe that immediate post-interventional loss of subjects in this study may be associated with acute adverse reactions.

The hypothesis of our study was conceived based on the findings of Sun and colleagues. They had suggested that HES could be used safely as an alternative to epidural blood patches [12]. Our results demonstrated a statistically significant effect of HES in comparison to controls; however, efficacy was not comparable to those seen in the PI, Talc and AB groups. Although statistical significance was not observed in the majority of comparisons between these 3 groups, the trends observed in our study indicate that the most effective agents were Talc and AB. In clinical practice, AB pleurodesis has gained widespread use for the treatment of persistent air leaks, especially in patients with spontaneous pneumothorax where the use of Talc cannot be considered due the possibility of severe adverse events. It is also important to note that AB has virtually no side effects compared to other chemical agents.

In this experimental study, although HES application caused significant variations from controls, our results do not support a reliable role for HES in pleurodesis.

### Limitations

The prominence of inflammation but the absence of comparable fibrosis may be interpreted as a limitation associated with the lack of sufficient time for the development of fibrosis. However, even though the study duration may be considered short in comparison to a few of the studies on this topic, the median duration of such studies is around 30 days. Furthermore, it is also apparent that the Talc and AB groups demonstrated significant effects within this 30-day period; thus the study duration was undoubtedly sufficient to test our hypothesis. Another limitation is the low number of subjects in each group, which was compounded by the loss of a total of 4 rats before planned sacrification. However, low number of subjects are a natural limitation in all animal-based studies, and the evaluation of previously published studies shows that the number of animals included in the final analysis were sufficient to draw conclusions.

## Conclusion

Even though our results did not show success with HES, we believe that future studies aimed at identifying better agents for pleurodesis will continue unabated due to the unreliable outcomes and adverse effects of available treatments. Easy access, low cost and good safety profile are probably the most crucial factors that will affect the search for an ideal pleurodesis agent. Even though HES application seems to be ineffective, our results indicate that the AB pleurodesis method remains as a safe, simple and cheap option that is considerably effective.

## Data Availability

The datasets generated and/or analyzed during the current study are not publicly available due to the use of a grant from COMUDAM, but are available from the corresponding author on reasonable request.

## Abbreviations

AB: Autologous blood
COMUDAM: Canakkale Onsekiz Mart University Experimental Research Application and Research Center
H&E: Hematoxylin & Eosin
HES: Hydroxyethyl Starch 130/0.4 (6%)
PI: Povidone-iodine
PTFE: Polytetrafluoroethylene
SF: Physiological Saline
